# Effect of ventilation strategy during cardiopulmonary bypass on postoperative pulmonary complications after cardiac surgery: a randomized clinical trial

**DOI:** 10.1186/s13019-021-01699-1

**Published:** 2021-10-30

**Authors:** Meng-Qiu Zhang, Yu-Qi Liao, Hong Yu, Xue-Fei Li, Wei Shi, Wei-Wei Jing, Zai-Li Wang, Yi Xu, Hai Yu

**Affiliations:** 1 Department of Anesthesiology, West China Hospital, Sichuan University and The Research Units of West China (2018RU012), Chinese Academy of Medical Sciences, Chengdu, 610041 China; 2 Department of Anesthesiology, The Third People’s Hospital of Chengdu, Chengdu, 610041 China

**Keywords:** Cardiac surgery, Cardiopulmonary bypass, Mechanical ventilation, Fraction of inspired oxygen, Postoperative pulmonary complications

## Abstract

**Background:**

To determine whether maintaining ventilation during cardiopulmonary bypass (CPB) with a different fraction of inspired oxygen (FiO_2_) had an impact on the occurrence of postoperative pulmonary complications (PPCs).

**Methods:**

A total of 413 adult patients undergoing elective cardiac surgery with CPB were randomly assigned into three groups: 138 in the NoV group (received no mechanical ventilation during CPB), 138 in the LOV group (received a tidal volume (V_T_) of 3–4 ml/kg of ideal body weight with the respiratory rate of 10–12 bpm, and the positive end-expiratory pressure of 5–8 cmH_2_O during CPB; the FiO_2_ was 30%), and 137 in the HOV group (received the same ventilation parameters settings as the LOV group while the FiO_2_ was 80%).

**Results:**

The primary outcomes were the incidence and severity of PPCs during hospitalization. The composite incidence of PPCs did not significantly differ between the NoV (63%), LOV (49%) and HOV (57%) groups (*P* = 0.069). And there was also no difference regarding the incidence of PPCs between the non-ventilation (NoV) and ventilation (the combination of LOV and HOV) groups. The LOV group was observed a lower proportion of moderate and severe pulmonary complications (grade ≥ 3) than the NoV group (23.1% vs. 44.2%, *P* = 0.001).

**Conclusion:**

Maintaining ventilation during CPB did not reduce the incidence of PPCs in patients undergoing cardiac surgery.

*Trial registration*: Chinese Clinical Trial Registry ChiCTR1800015261. Prospectively registered 19 March 2018. http://www.chictr.org.cn/showproj.aspx?proj=25982

**Supplementary Information:**

The online version contains supplementary material available at 10.1186/s13019-021-01699-1.

## Background

Every year, over 1.25 million patients undergo cardiac surgery utilizing cardiopulmonary bypass (CPB) globally, with approximately 180,000 of those operations conducted in China [1]. Postoperative pulmonary complications (PPCs), including pneumonia, pleural effusion, atelectasis, etc., are common, with reported incidence of up to 59.2% [2]. These well-documented complications prolong the hospital stay and increase in-hospital mortality after surgery [3].

Attempts concentrating on lung-protective ventilation strategies have been made to prevent PPCs. The intraoperative lung-protective ventilation bundle has been identified as an independent association with reduced PPCs after cardiac surgery [4]. Mechanical ventilation with low tidal volume (V_T_) during CPB appears to be beneficial [5], though conflicting data exist [6, 7]. A recent meta-analysis indicated that the ventilation during CPB may improve the gas exchange and oxygenation index (OI), while the long-term outcomes are unknown. And the settings of the fraction of inspired oxygen (FiO_2_) may make a difference [8]. Moreover, recently published guidelines on CPB in adult cardiac surgery recommend that "PEEP during CPB should be considered in order to protect the lungs" and "Ventilation during CPB may be considered for lung protection" (level of evidence B) [9]. In addition, the arguments both for and against the use of hyperoxia in cardiac surgery are still ongoing [10, 11]. However, the effect of low V_T_ ventilation with different FiO_2_ during CPB in patients having cardiac surgery is still unclear.

Therefore, we conducted this pragmatic randomized trial to investigate the effect of ventilation with different FiO_2_ or suspended ventilation during CPB on the occurrence of PPCs in patients undergoing cardiac surgery.

## Methods

### Trial design and overview

This prospective randomized, controlled, patient-and-evaluator-blinded trial was implemented over the period from May 2018 to March 2019. It was approved by the Ethics Committee of West China Hospital of Sichuan University and registered before participant enrolment at the Chinese Clinical Trial Registry (ChiCTR1800015261). Written informed consent was obtained from each participant in the ward before the day of surgery. The study protocol has been published elsewhere [12]. This study combined different ventilation strategies and different FiO_2_. Briefly, the patients were randomized into three groups: (1) the NoV group: no ventilation during CPB; (2) the LOV group: participants received a low V_T_ of 3–4 ml/kg of ideal body weight (IBW) with the respiratory rate (RR) of 10–12 bpm, and the positive end-expiratory pressure (PEEP) of 5–8 cmH_2_O during CPB; the FiO_2_ is 30% with a flow of 2 L/min; (3) the HOV group: the FiO_2_ is 80% with a flow of 2 L/min; the ventilation parameter settings were same as the LOV group.

### Study population

Patients undergoing elective cardiac surgery (age ≥ 18 years old) with CPB were recruited in the trial. Patients having pregnant and those who have long-term use of hormone therapy before surgery (≥ 3 months) or have already participated in other similar researches within 3 months were excluded from the participation. Heart transplantation, pulmonary thromboendarterectomy, aortic arch, and other deep hypothermic circulatory arrest surgeries and concomitant other non-cardiac interventions were also excluded.

### Randomization and blinding

According to the randomization list generated by Microsoft Excel, all patients were allocated in a 1:1:1 treatment ratio to one of the three groups: (1) the NoV group; (2) the LOV group; (3) the HOV group. One investigator not involved in the trial was responsible for screening and grouping the patients. He reviewed the patients’ hospitalized records on the day before surgery, interviewed patients who met the criteria, and signed informed consent. He would also pass the opaque, sealed, and sequentially numbered envelopes to the attending anesthesiologists, who were in charge of patients’ treatments. The attending anesthesiologists and surgeons were not blinded due to the characteristics of the study. The patients, the data collectors, evaluators and the intensivists, respiratory therapists (RTs) were unaware of the allocation and intervention information.

### Anesthesia and perioperative care

No pre-medication was administered. Intraoperative monitoring included five-lead electrocardiogram, invasive artery blood, central venous pressure, pulse oxygen saturation, end-tidal carbon dioxide partial pressure (P_ET_CO_2_), transesophageal echocardiogram, nasopharyngeal temperature, and bispectral index (BIS).

Induction medication was decided by the attending anesthesiologists. Preoxygenation for denitrogenation was administrated before endotracheal intubation. Continuous infusing remifentanil, combined with sevoflurane and/or propofol to maintain the BIS level between 40 and 60. After intubation, volume-controlled ventilation strategy was applied with the following settings: V_T_ = 6–8 ml/kg IBW, PEEP = 5–8 cmH_2_O, I/E ratio = 1:2, FiO_2_ = 50%, and RR = 10–12 bpm, maintaining P_ET_CO_2_ between 35 and 45 mmHg, intermittent vital capacity maneuvers (VCMs) were carried out with manual ventilation creating the airway pressure at 30 cmH_2_O for at least 5 s at the end of CPB and before transferring out of the operation room. Heparin 3 mg/kg was given intravenously to obtain an activated clotting time (ACT) of 480 s to start the CPB. PaO_2_ during CPB was maintained between 150 and 250 mmHg and mean arterial blood pressure was maintained between 50 and 70 mmHg. At the end of CPB, heparin was antagonized by protamine in a dose of 1:1 to bring ACT to baseline levels.

Synchronized intermittent mandatory ventilation was applied immediately after patients were transferred to the ICU. The ventilation parameter settings were as follow: V_T_ = 6–8 ml/kg IBW, PEEP = 8 cmH_2_O, RR = 12 bpm, FiO_2_ = 50%. FiO_2_ was adjusted according to the PaO_2_ by performing arterial blood gas analyses. FiO_2_ was decreased by 10% if the PaO_2_ was higher than 150 mmHg, increased by 10% if lower than 80 mmHg. The minimum FiO_2_ was 30%. Other therapeutic approaches and decisions were all up to the ICU physicians.

### Data collection

Data were independently collected by a researcher who was blinded to treatment assignment and also not involved in the clinical care decision making. Baseline characteristics included gender, age, body mass index, smoking history, pulmonary diseases, hypertension, and diabetes mellitus. Intraoperative data included types and duration of surgery, duration of CPB, and aortic cross-clamp (ACC), allogeneic red blood cell unit. Postoperative data included the intubation time and the length of ICU and hospital stay.

### Outcomes

The primary outcomes were the composite incidence of the PPCs [13] (Additional file 1: Table S1) and the PPCs severity score [14] (Additional file 1: Table S2) during the hospital stay. Chest radiographs were obtained for all patients in the first 24 h after surgery in addition to those requested by the ICU physician. The occurrence and severity of PPCs were assessed daily, until hospital discharge, using the worst score during the hospital stay for the main analyses. Each postoperative pulmonary complication, the worst patients experienced throughout their hospital stay, was graded from 1 to 5. The higher the grade was, the worse the condition. Pulmonary complications were defined according to symptom, sign, and chest imaging examination. Patients with symptoms of pulmonary complications were examined by chest radiographs or CT according to clinical practice by the attending physician. All chest imaging was evaluated by the blinded attending radiologist. Arterial blood gas analyses would be administrated when shortness of breath, dyspnea, or SpO_2_ < 92% occurs. The complications were measured by outcome evaluators and surgeons together. The second outcomes were OI (calculate as: OI = PaO_2_/FiO_2_), surgical site infection, intubation time, length of ICU stay, hospital stay, and in-hospital and 30-d mortality. The occurrence of OI < 300 mmHg was compared at three time points: the moment arriving in the ICU, 6, and 12 h after arrival in the ICU.

### Statistical methods

Due to the limited data on the effect of ventilation strategies during CPB on PPCs when the study was designed, we conducted a pilot study to preliminarily observe the incidence of PPCs in our center. The sample size was determined to obtain 80% power to show an 18-point absolute difference in the occurrence of PPCs during hospital stay between the two groups (40% in the NoV group vs. 22.2% in the LOV group, based on the pilot study). We calculated that 127 patients per group were needed with a 2-sided α = 0.05. To allow for 10% attrition, 420 patients were needed (140 patients per group).

Outcomes data were analyzed in the intention-to-treat (ITT) population. The Kolmogorov–Smirnov and Levene’s test were used to analyse the normality and variance homogeneity. The normally distributed continuous data satisfying the homogeneity of variance were presented as mean [standard deviation (SD)] and compared using one-way analysis of variance (ANOVA). Fisher's least significant difference-t test (LSD-t test) was used for post hoc analysis. The non-normally distributed and ordinal data were presented as median (inter-quartile range) and compared using the non- parametric Kruskal–Wallis test. When significant differences were found, the test was followed by pairwise comparison using the Manne-Whitney U-test, with Bonferroni adjustment for multiple tests (three comparisons). The categorical data were presented as the number (percentage) and compared using the Pearson χ^2^ test or Fisher's exact test. Statistical analyses were accomplished with SPSS 20.0 (IBM, Chicago, IL, USA).

To maintain an overall familywise error rate of less than 0.05, the threshold for type I error rate was adjusted for each of the two co-primary outcomes to 0.025 (2-sided). Furthermore, a Bonferroni-adjusted *P*-value < 0.0083 (0.025/3) was considered statistically significant to control type I error for the pairwise comparisons between the three groups [15].

## Results

### Study population

**Fig. 1:**
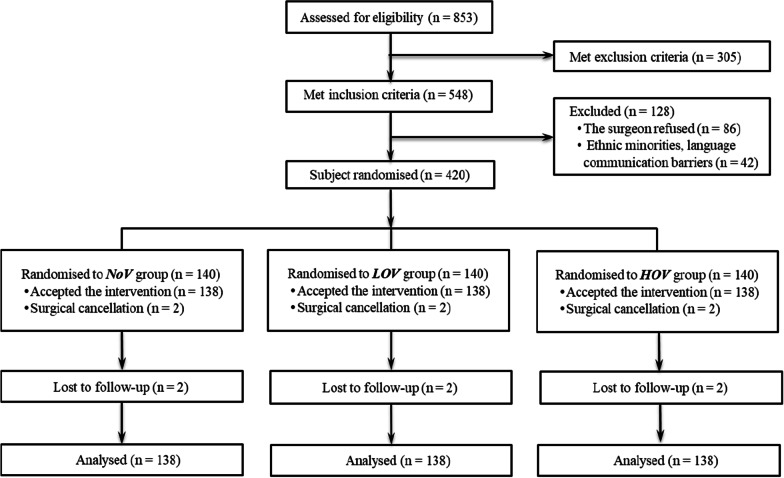
Patient randomization, follow-up, and analysis populations

Enrolment ceased when the target sample size was obtained. A total of 420 patients were randomized in the study and 140 patients were randomized into each group initially. Seven patients were excluded because of the cancellation of surgery. Therefore, 413 patients completed the study and were included in the data analyses (Fig. 1). There were no significant differences in baseline characteristics and intraoperative variables among the three groups (Table 1).

**Table 1:** Baseline characteristics and surgical data

	NoV (n = 138)	LOV (n = 138)	HOV (n = 137)	*P* value
Age (year)	53 (46–61)	52 (45–63)	52 (46–61)	0.796
Gender (M/F), n	62/76	64/74	67/70	0.800
Smoking, n (%)	31 (22.5)	30 (21.7)	31 (22.6)	0.982
BMI	23.1 (20.7–25.2)	22.6 (20.7–25.1)	23.2 (20.9–25.1)	0.650
ASA III, n (%)	134 (97.1)	137 (99.3)	136 (99.3)	0.339
EuroScore II	1.07 (0.84–1.39)	1.07 (0.86–1.51)	1.02 (0.86–1.35)	0.683
Preoperative OI	385 (376–404)	385 (365–404)	385 (371–409)	0.485
Comorbidities, n (%)				
COPD	3 (2.2)	6 (4.3)	5 (3.6)	0.629
Hypertension	23 (16.7)	18 (13.0)	22 (16.1)	0.669
Diabetes	5 (3.6)	7 (5.1)	12 (8.8)	0.172
Prior stroke	3 (2.2)	3 (2.2)	5 (3.6)	0.693
Surgical type, n (%)				
Valve replacement/repair	116 (84.1)	113 (81.9)	120 (87.6)	0.419
CABG	11 (8.0)	11 (8.0)	9 (6.6)	0.878
Repair of structural defect	16 (11.6)	23 (16.7)	17 (12.4)	0.418
Resection of a cardiac tumour	1 (0.7)	3 (2.2)	3 (2.2)	0.643
Anesthetics administration				
Sufentanil (ug/kg)	2.7(2.2–3.5)	3.3(2.6–4.1)	2.9(2.5–3.9)	0.661
Cisatracurium (mg/kg)	0.6(0.5–0.8)	0.7(0.5–0.9)	0.7(0.4–0.8)	0.856
Duration of surgery (min)	235 (196–291)	240 (203–280)	245 (210–286)	0.851
Duration of CPB (min)	116 (93–140)	117 (95–155)	123 (90–154)	0.737
Duration of ACC (min)	77 (65–105)	83 (60–110)	83 (60–113)	0.661
ACC ≥ 90 min, n (%)	62 (44.9)	50 (36.2)	60 (43.8)	0.281
Application of NIV in ICU, n (%)	51 (36.9%)	64 (46.4%)	47 (34.3%)	0.098
Intraoperative infusion (L)	1.2 (1.0–1.4)	1.2 (1.0–1.5)	1.2 (0.9–1.5)	0.532
Allogeneic RBC (u)	2 (0–6)	2 (0–8)	1 (0–8)	0.121

*BMI* body mass index, *ASA* American society of anesthesiologists, *OI* oxygenation index, *COPD* chronic obstructive pulmonary disease, *CABG* coronary artery bypass graft, *CPB* cardiopulmonary bypass, *ACC* aortic crossclamp, *NIV* non-invasive ventilator, *ICU* intensive care unit, *RBC*: red blood cells

### Primary outcomes

**Fig. 2:**
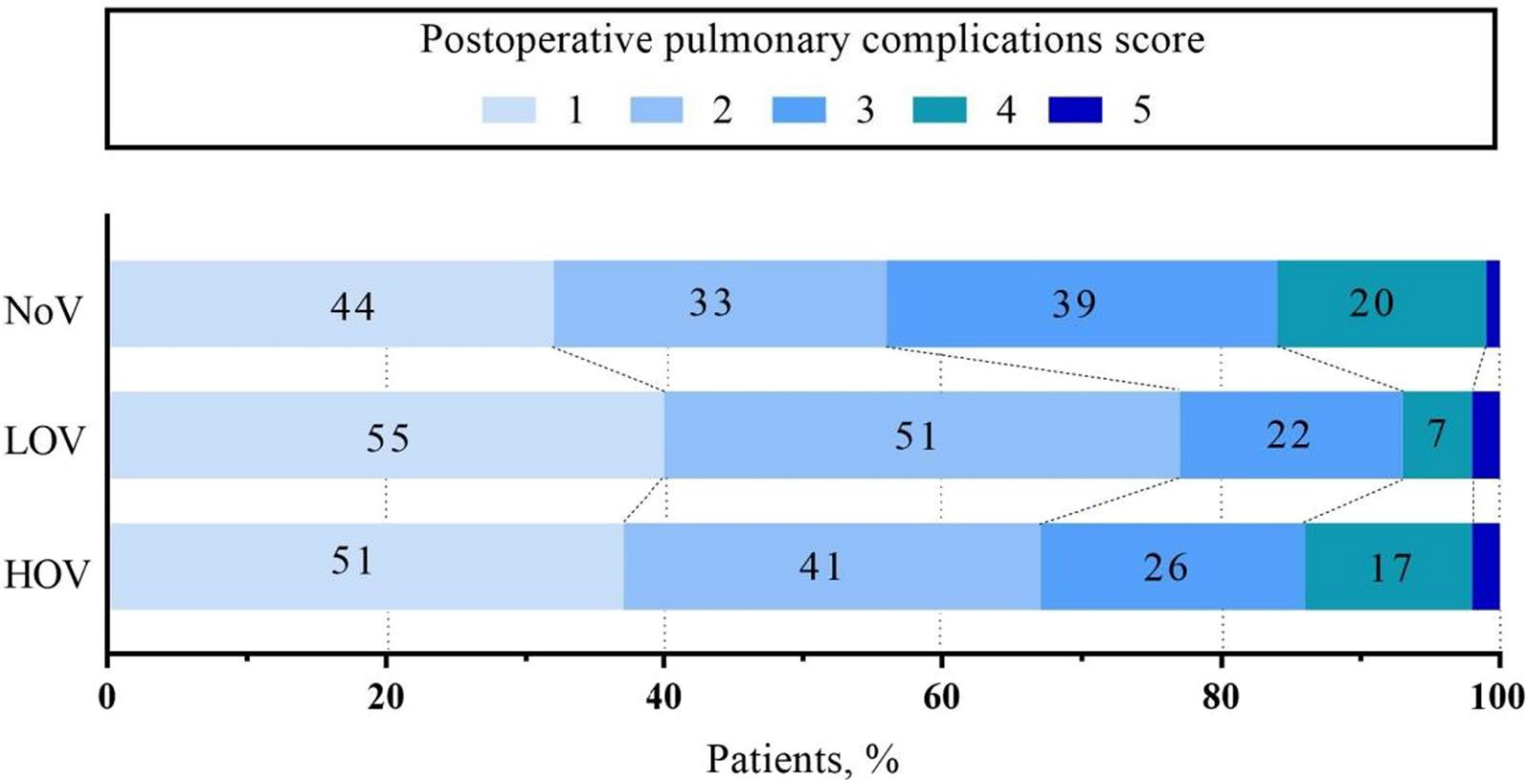
Severity of postoperative pulmonary complications

Patients who experienced at least one episode of respiratory failures, respiratory infection, pleural effusion, atelectasis, aspiration pneumonitis, bronchospasm, or pneumothorax or any combination of these during hospital stay were considered to have PPCs. The composite incidence of PPCs was 63% in the NoV group, 49%in the LOV group, and 57% in the HOV group (*P* = 0.069; Table 2). Also, no difference was observed for this outcome in the combined ventilation groups compared with that in the no ventilation group (53% versus 63%; odds ratio [OR], 0.66; 95% confidence interval [CI], 0.44–1.01; *P* = 0.054). There was also no statistical difference among the three groups in the comparison of each complication event (Table 2). The incidence of moderate and severe pulmonary complications (grade ≥ 3) that occurred in LOV group was significantly lower than that in NoV group (*P* = 0.001) (Fig. 2).

**Table 2:** Outcomes

	NoV (n = 138)	LOV (n = 138)	HOV (n = 137)	*P* value
Primary outcome				
Composite incidence, n (%)	87 (63)	68 (49)	78 (57)	0.069
Pleural effusion (needing thoracentesis)	46 (33)	39 (28)	32 (23)	0.185
Respiratory failure	21 (15)	19 (14)	17 (12)	0.796
Respiratory infection	29 (21)	20 (15)	25 (18)	0.366
Pulmonary atelectasis	22 (16)	19 (14)	32 (23)	0.092
Pneumothorax	5 (4)	2 (2)	3 (2)	0.490
Aspiration pneumonia	0	0	0	–
Bronchospasm	0	0	0	–
Secondary outcomes				
Surgical site infection, n (%)	4 (2.9)	3 (2.2)	2 (1.5)	0.913
ICU intubation time (h)	14.5 (8.4–35.3)	15.5 (9.0–37.3)	16.0 (9.0–35.0)	0.896
Duration of ICU (d)	3 (2–4)	3 (2–4)	3 (2–4)	0.697
Hospital stay (d)	8 (7–10)	8 (6–10)	8 (6–10)	0.837
Hospital mortality, n (%)	4 (2.9)	3 (2.2)	2 (1.5)	0.913
30-d mortality, n (%)	5 (3.6)	4 (2.9)	4 (2.9)	0.701

### Other outcomes

**Fig. 3:**
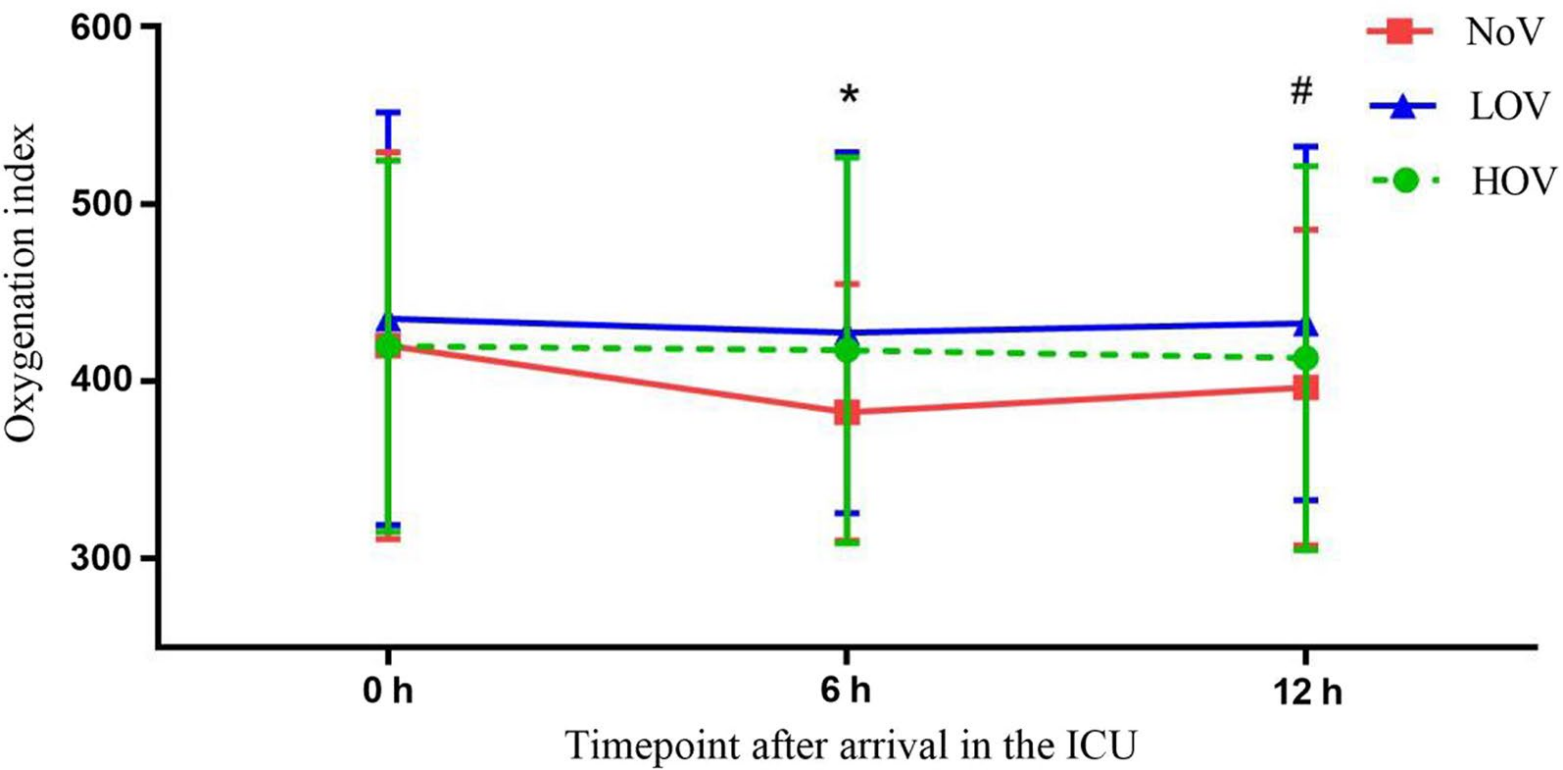
Comparison of oxygenation index. (*LOV vs. NoV, *P* = 0.003; #LOV vs. NoV, *P* = 0.016)

In the comparison of OI among the three groups in the time points of 6 h and 12 h after arrived ICU. The LOV group was superior to the NoV group in those two time points (*P* = 0.003 and 0.016, respectively) (Fig. 3). There was no significant difference in surgical site infection, ICU intubation time, ICU stay, hospital stay, and in-hospital and 30-d mortality among the three groups (Table 2).

## Discussion

This study revealed that continuation of low V_T_ ventilation with 30% or 80% FiO_2_ were not superior to no ventilation during CPB, with respect to the incidence of PPCs during hospital stay after surgery. However, given the study design, it was also difficult to draw strong conclusions on the application of low V_T_ ventilation with 30% FiO_2_ may reduce the severity of PPCs.

The lungs are particularly vulnerable during cardiac surgery. Adequate intraoperative pulmonary management and monitoring are paramount in preventing post-cardiac pulmonary complications. However, the traditional practice is to suspend ventilation during CPB for the best exposure of the surgical field [16]. The goal of this trial was to investigate whether the use of low V_T_ ventilation with different level of FiO_2_ or not during CPB has an influence on PPCs. The composite incidence of PPCs in this study is relatively high, which only reflects the situation of our single medical center. Maintaining low V_T_ ventilation combined with different FiO_2_ during CPB did not reduce the incidence of PPCs. The incidence of PPCs in our study is 56%. With similar findings, the incidence of PPCs in the PROVECS trial was also high (59.2%) [2]. That study found that maintaining mechanical ventilation with higher PEEP level during CPB did not reduce PPCs, as compared with a conventional strategy with no ventilation during CPB and lower PEEP level. And all the participants were received a 40% FiO_2_ during CPB. It is the first prospective study to assess pulmonary complications, which is highly relevant to clinical practice. The incidence of PPCs in that trial is similar to our LOV group’s, probably due to the similarity of design.

The top three incidences of PPCs in our study were pleural effusion, atelectasis, and pulmonary infection, which is consistent with the results of a previous study [17], while that in the PROVECS trial were respiratory failure, respiratory acidosis, and radiological atelectasis [2]. The surgical type in our study is mainly valvular procedure (over 80%) due to rheumatic heart disease, which is much higher than the PROVECS’s. And the subjects in that study were older than our subjects. These may lead to a difference in the incidence of PPCs.

In the present study, the proportion of moderate and severe pulmonary complications in the LOV group was 21% lower than that in the NoV group (23% vs. 44%; OR, 0.38; 95% CI, 0.23–0.64; *P* = 0.001). It indicated that a low V_T_ ventilation strategy with 30% FiO_2_ may attenuate the severity of pulmonary complications. Previous studies have not used this parameter as a primary comparison indicator. Similar to that result, the LOV group had higher OI at 6 and 12 h after arrival in the ICU than the NoV group. This result is consistent with Beer’s study [18], in which the participants received a V_T_ of 3–4 ml/kg ventilation during CPB. They found that the ventilated group has higher OI than the unventilated group at the end of the operation and 6 h, 12 h after arrival in the ICU. Even if there was no difference in postoperative ventilator support time between the two groups, they still suggested that maintaining ventilation during CPB was superior in reducing postoperative lung injury. However, the increased OI did not influence the incidence of PPCs or other outcomes, such as intubation time and in-hospital mortality in our study. A lower incidence of moderate to severe PPCs in the LOV group as compared to the NOV and HOV groups as well as better OI primarily at 6 h after surgery, thus implying a biphasic effect of FiO_2_. Determining whether low V_T_ ventilation with different FiO_2_ during CPB provides clinical benefit in cardiac surgical patients is challenging, because procedure-related pulmonary complications may obscure the benefits from a relatively brief duration of mechanical ventilation during CPB. The efficacy of low V_T_ ventilation combined with different FiO_2_ during CPB shows here, together with the low effort and zero costs necessary for implementation and with no side effects observed or expected, makes it hard to argue against using this simple method for reduction of PPCs.

The most recently published, MECANO trial recruited a large number of patients (n = 1501). No differences were observed between the VENT group (5 bpm with a V_T_ of 3 ml/kg and PEEP of 5 cmH_2_O) and the control group (no ventilation) in the hospital mortality, early respiratory failure, ventilation support beyond day 2 and reintubation. Unlike our study, more than half of the types of surgery are CABG (62%). Yet, the results were similar to our study, without any difference regarding clinical outcomes between the two groups, such as PPCs and in-hospital mortality [19].

There are also some limitations to this study. First, this was a single-center study, all of the perioperative management were carried out according to our hospital’s clinical practice. Second, in the type of surgery, the majority of patients (> 80%) underwent valvular surgery due to rheumatic heart disease. Therefore, the conclusion drawn by our study should be carefully applied to other medical centers. Third, such a difference between expected and observed incidence of pulmonary complications may have implications in the required sample size and study power. Notably, absolute risk reduction is 14%, and a relative risk reduction of 22% with a no costs intervention are significant findings and should not be diminished by the firm conclusions. The use of composite outcomes also offers the interest to reduce sample sizes; however, it may be responsible for difficulties in the interpretation of the results. Nonetheless, for each component included in our composite outcome, the differences in the degree of severity and the incidence had been compared. Fourth, we only applied the volume-controlled ventilation mode in the study. It is also unclear that which ventilation mode (volume-controlled or pressure-controlled ventilation) is superior, though a previous study suggested there is no difference between them [20]. Fifth, in consideration of maintaining hemodynamic stability, administering VCMs was not standard as reported in the literature [21], but we can ensure the effectiveness of the maneuvers for reopening the collapsed lung. We also didn’t record the detailed usage of inotrope, left ventricular function, and other intraoperative ventilation-related parameters such as peak inspiratory pressure. Whether there is a difference in those patients is unclear. Given that the management of the ICU were carried out by the intensivists and RTs, the effects of our interventions may be obscured by the supplementary oxygen therapies and prolonged ventilation time.

## Conclusion

In conclusion, our study showed that maintaining low V_T_ ventilation combined with different FiO_2_ during CPB did not reduce the incidence of PPCs in comparison to the conventional strategy with no ventilation. Though a difference was not confirmed and no clinically relevant difference was observed, the application of low V_T_ ventilation with 30% FiO_2_ during CPB may have a trend to attenuate the severity of PPCs.

## Supplementary Information

**Additional file 1**. Protocol of VONTCPB.

## Data Availability

Data are available upon request.

## Abbreviations

ACT: Active clotting time
ACC: Aortic cross clamp
ANOVA: Analysis of variance
BIS: Bispectral index
CPB: Cardiopulmonary by pass
FiO_2_: Fraction of inspired oxygen
IBW: Ideal body weight
ITT: Intention-to-treat
OI: Oxygenation index
PPCs: Postoperative pulmonary complications
PaO_2_: Partial pressure of artery oxygen
PEEP: Positive end-expiratory pressure
P_ET_CO_2_: End-tidal carbon dioxide partial pressure
RCTs: Randomized controlled trials
RR: Respiratory rate
SD: Standard deviation
VCMs: Vital capacity maneuvers
